# Tomato and lemon extracts synergistically improve cognitive function by increasing brain-derived neurotrophic factor levels in aged mice

**DOI:** 10.1017/S0007114523002301

**Published:** 2024-04-14

**Authors:** Kyeong-No Yoon, Yidan Cui, Qing-Ling Quan, Dong Hun Lee, Jang-Hee Oh, Jin Ho Chung

**Affiliations:** 1 Department of Biomedical Sciences, Seoul National University Graduate School, Seoul, Republic of Korea; 2 Laboratory of Cutaneous Aging Research, Biomedical Research Institute, Seoul National University Hospital, Seoul, Republic of Korea; 3 Institute of Human-Environmental Interface Biology, Medical Research Center, Seoul National University, Seoul, Republic of Korea; 4 Department of Dermatology, Seoul National University College of Medicine, Seoul, Republic of Korea; 5 Institute on Aging, Seoul National University, Seoul, Republic of Korea

**Keywords:** Tomato and lemon, Cognitive function, Neurogenesis, Brain-derived neurotrophic factor

## Abstract

Brain ageing, the primary risk factor for cognitive impairment, occurs because of the accumulation of age-related neuropathologies. Identifying effective nutrients that increase cognitive function may help maintain brain health. Tomatoes and lemons have various bioactive functions and exert protective effects against oxidative stress, ageing and cancer. Moreover, they have been shown to enhance cognitive function. In the present study, we aimed to investigate the effects of tomato and lemon ethanolic extracts (TEE and LEE, respectively) and their possible synergistic effects on the enhancement of cognitive function and neurogenesis in aged mice. The molecular mechanisms underlying the synergistic effect of TEE and LEE were investigated. For the *in vivo* experiment, TEE, LEE or their mixture was orally administered to 12-month-old mice for 9 weeks. A single administration of either TEE or LEE improved cognitive function and neurogenesis in aged mice to some extent, as determined using the novel object recognition test and doublecortin immunohistochemical staining, respectively. However, a significant enhancement of cognitive function and neurogenesis in aged mice was observed after the administration of the TEE + LEE mixture, which had a synergistic effect. *N*-methyl-d-aspartate receptor 2B, postsynaptic density protein 95, and brain-derived neurotrophic factor (BDNF) levels and tropomyosin receptor kinase B (TrkB)/extracellular signal-regulated kinase (ERK) phosphorylation also synergistically increased after the administration of the mixture compared with those in the individual treatments. In conclusion, compared with their separate treatments, treatment with the TEE + LEE mixture synergistically improved the cognitive function, neurogenesis and synaptic plasticity in aged mice via the BDNF/TrkB/ERK signalling pathway.

Ageing declines the cognitive function including memory, processing speed, information retrieval and attention. Therefore, maintaining cognitive function during ageing is an important research topic^(1)^. Neurogenesis, the generation of functional neurons, is a process of brain plasticity involved in learning and memory^(2)^ and is also essential for cognitive memory. Maintenance of cognitive function is intricately linked to neurogenesis. In particular, as the primary brain area responsible for cognitive function^(3)^, the hippocampus is involved in the formation of memory and plays a crucial role in the regulation of cognition in adults^(4)^. The hippocampus consists of the dentate gyrus (DG) and cornu ammonis (CA), including CA1–CA3. Hippocampal neurogenesis occurs only in the DG, thereby making it a distinctive feature of this hippocampus area^(5)^. Neurogenesis is affected by various factors that occur during ageing, such as oxidative stress and inflammation^(2)^. As ageing progresses, hippocampal volume declines, neurogenesis diminishes and memory deficits begin to appear^(6)^.

Previous studies have demonstrated that ageing of the brain is associated with a decline in synaptic plasticity^(7)^. Synaptic plasticity and synaptogenesis are necessary for learning and memory^(8)^. Thus, the measurement of synaptic plasticity is an important indicator for evaluating cognitive function. Postsynaptic density protein-95 (PSD-95), synaptophysin (SYP) and *N*-methyl-d-aspartate receptor (NMDAR) are widely used markers for assessing synaptic plasticity and synaptogenesis^(9,10)^. PSD-95 is a member of the membrane-associated guanylate kinase class of postsynaptic proteins. It plays a major role in synaptic maturation and function^(11)^. SYP is a calcium-binding hexametric tyrosine-phosphorylated protein that is extensively distributed in the presynaptic vesicle membrane and plays a crucial role in synaptic formation and vesicular endocytosis^(12)^. NMDAR is localised in the postsynaptic density and is an important glutamate receptor associated with synapse formation. The NMDAR signalling pathway in postsynaptic neurons plays an important role in regulating learning and memory^(13)^.

Brain-derived neurotrophic factor (BDNF) plays a crucial role in neurogenesis (especially hippocampal neurogenesis) and the maintenance of cognitive function. It is an important neurotrophic factor that regulates learning and memory by regulating synaptic plasticity in the brain via the activation of signalling pathways and neuronal growth and differentiation^(14)^. Increased levels of BDNF have been detected in various brain regions such as the hippocampus, cerebral cortex, amygdala and cerebellum, with the highest level detected in hippocampal neurons^(15)^. However, evidence suggests that BDNF levels decrease with age in both mice and humans. Several studies have reported that BDNF protein and mRNA levels decline with age in various brain regions, including the hippocampus and cortex^(16)^. Hippocampal BDNF levels are also reported to decline during states of psychological stress combined with obesity, as well as myocardial BDNF levels^(17)^. Hippocampal neurogenesis requires high levels of BDNF, and its exogenous application can stimulate this process^(18)^. BDNF is synthesised as pro-BDNF and cleaved into mature BDNF by extracellular proteases, such as metalloproteinases and plasmin^(19)^. Mature BDNF binds to the tropomyosin receptor kinase B (TrkB) receptor, which increases neuronal survival, neuroplasticity and synaptogenesis through extracellular signal-regulated kinase 1/2 (ERK1/2)/cyclic AMP response element-binding (CREB) signalling pathways^(20)^. BDNF/TrkB signalling is also known to be essential for proper myocardial function^(21)^, and it is known that patients with cardiac disease have a high prevalence of cognitive impairment^(22)^. Thus, BDNF has been implicated as a biomarker and drug target for neurodegenerative and neuropsychiatric disorders, as it plays multiple roles in various neuronal functions in the central nervous system^(23)^.

Tomato (*Lycopersicon esculentum*) is a rich source of folic acid, vitamin C and potassium and contains bioactive constituents with positive health effects, including antioxidant and anticancer effects^(24)^. Oral administration of tomato extract improves cognitive function through hippocampal neurogenesis in aged mice^(25)^. Aqueous extracts of tomato seeds have been shown to exert neuroprotective effects against mitochondrial dysfunction^(26)^. Lemon (*Citrus limon*) is a rich source of vitamin C, flavonoids, phenolic compounds and carotenoids and contains bioactive constituents with antioxidant, anticancer and anti-inflammatory effects^(27)^. Lemon essential oil improved the cognitive function in amyloid precursor protein/presenilin-1 double transgenic Alzheimer’s disease mice with cognitive impairment by inhibiting acetylcholinesterase^(28)^. Recently, it was reported that a mixture of tomatoes and lemons had synergistic effects on hippocampal neurogenesis and anti-oxidative stress^(29)^. However, the mechanism underlying this synergism has not been studied and requires further elucidation. Thus, in the present study, we aimed to identify the mechanisms associated with the synergistic activity of the tomato and lemon extracts against cognitive decline.

## Experimental methods

### Preparation of tomato ethanolic extract and lemon ethanolic extract

Dried tomato powder (50 kg) was extracted using 500 l of 70 % ethanol at 80°C for 6 h. The extract was filtered (1 μm) and concentrated using a rotary evaporator at 10–15 °brix. The resulting extract was mixed with dextrin at a ratio of 50wt% solid content in the tomato extract and 50wt% dextrin and then sterilised at 80°C for 40 min. It was spray-dried to produce the tomato extract powder (5·6 kg). Dried lemon peel (50 kg) was extracted twice. The first extraction was performed by adding 500 l of 70 % ethanol at 80°C for 4 h, and the second extraction was performed with the residue by adding 500 l of 70 % ethanol at 80°C for 2 h. The extract was filtered (1 μm) and concentrated using a rotary evaporator at 10–15 °brix. The resulting extract was mixed with dextrin at a ratio of 50wt% solid content in lemon peel extract and 50wt% dextrin and then sterilised at 80°C for 40 min. It was spray-dried to obtain the lemon peel extract powder (9·1 kg). The tomato and lemon peel extract powders were used in the experiment, mixed at a ratio of 1:1.

### Animal preparation for the experiment

Twelve-month- and eight-week-old male C57BL/6 mice (aged and young mice, respectively) were purchased from the Korea Institute of Basic Science and Support (Gwangju, Korea). The mice were allowed *ad libitum* access to food and were adapted for a week before the study. All experimental protocols were approved by the Institutional Animal Care and Use Committee (case number: 20–0270-S1A2) of the Biomedical Research Institute at Seoul National University Hospital and were performed in accordance with the relevant guidelines and regulations.

### Animal grouping and oral administration of tomato ethanolic extract and lemon ethanolic extract

**Fig. 1.**
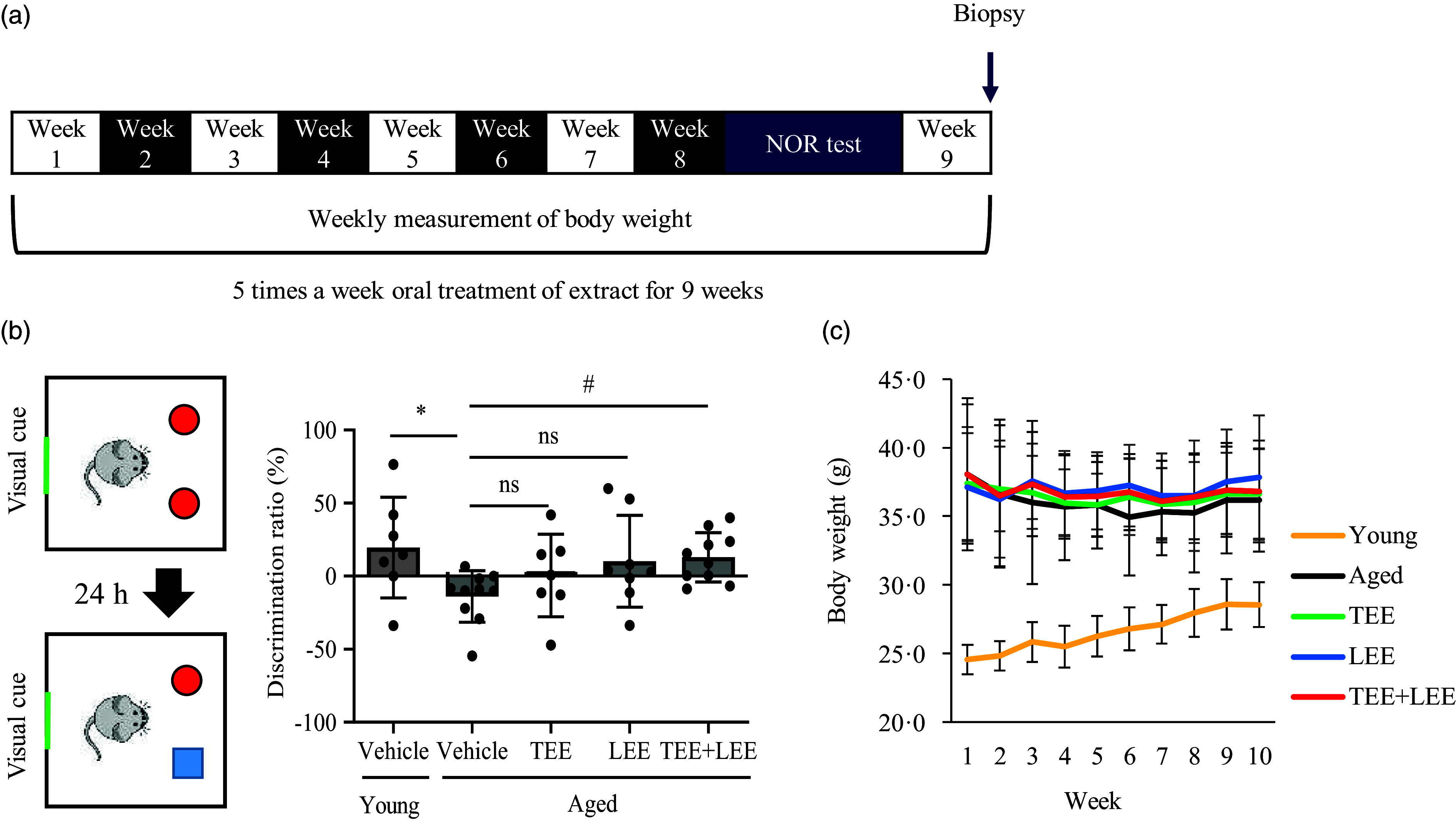
Oral administration of tomato (TEE) and lemon ethanolic extract (LEE) synergistically improved age-related memory impairments in aged mice. (a) Twelve-month-old and eight-week-old mice were orally administered the extracts or vehicle, respectively, 5 d a week for 9 weeks. (b) After 8 weeks of oral administration, the novel object recognition (NOR) test was performed. After oral administration of TEE, LEE and their mixture to aged mice, their preferences for exploring new objects were identified using the NOR test (young + vehicle, *n* 7 mice; aged + vehicle, *n* 9 mice; aged + TEE, *n* 7 mice; aged + LEE, *n* 8 mice; aged + TEE and LEE, *n* 10). The discrimination index was calculated as the difference between the exploration time for the novel object (N) and that of the familiar object (F) divided by the total time spent exploring both objects (discrimination index = (N − F)/(N + F)). (c) Changes in mouse weight during the study period. Each bar represents the mean ± se for each group. Statistical significance was determined by Kruskal–Wallis *H* test followed by post hoc Dunn’s test. *P < 0·05 *v*. young vehicle group. ^#^ *P* < 0·05 *v*. the aged vehicle group.

The aged mice were randomly divided into four groups (*n* 10 each): three extract-intake groups and a vehicle control group. Once a day for 9 weeks, the mice in the three treatment groups were orally administered TEE (200 mg/kg), LEE (200 mg/kg) or their equal mixture (TEE 200 mg/kg + LEE 200 mg/kg) using feeding needles. Vehicle-treated young and aged mice were administered an equal volume (100 µl) of 0·5 % carboxymethyl cellulose-sodium solution (vehicle) once daily for 9 weeks. The mice were sacrificed 1 d after the last dose (Fig. 1(a)).

### Novel object recognition test

The novel object recognition test was carried out at 8 weeks after oral administration of the extract. Before the novel object recognition test, the mice were accustomed for 5 min a day for 4 d to reduce anxiety. The mice were acclimatised in a black plastic box (32 cm × 32 cm × 32 cm). After placing two objects of the same material, shape, colour and size in the box, the time of interest in each object was measured for 10 min. Mice with object bias were excluded from the experiment. After 24 h, one object was replaced with a new object, and time of interest was measured for 5 min. The new object was different from the existing object only in shape and size, and it was considered that a mouse was interested in the object when it touched the object with its nose or paw. Object recognition ability was determined by measuring the time required to search for a new material and obtaining a ratio to the total search time, that is a discrimination index.

### Brain sample collection

All mice were anaesthetised using isoflurane, and the brains were carefully removed from the mice. The removed brains were fixed in 4 % paraformaldehyde solution overnight at 4°C. Following fixation, the brains were immersed in a solution of 30 % sucrose in 0·05 M phosphate-buffered saline. The fixed and cryopreserved brains were cut into sequential coronal sections using a freezing microtome. The sections were 35-μm-thick, which means each slice was approximately 35 micrometres thick. The sections were stored in a cryoprotectant solution containing 25 % glycerol in 0·05 M phosphate-buffered saline at 4°C until the immunohistochemistry procedure was performed.

### Immunohistochemistry

Doublecortin (DCX) and p-TrkB (S478) immunostaining were performed using a free-floating technique. Randomly selected sections were incubated for 2 d at 4°C with DCX antibody (sc-8066, Santa Cruz Biotechnology, Santa Cruz, CA, USA, 1:200) and p-TrkB (R-1718–50, Biosensis, Thebarton, Australia, 1:100) in a dilution buffer containing 1 % bovine serum albumin (Sigma-Aldrich, St. Louis, MO, USA) and 1 % Triton X-100 in 0·1 M phosphate buffer. After washing with phosphate-buffered saline buffer, the sections were reacted with horseradish peroxidase-conjugated anti-goat or rabbit IgG secondary antibody (Vector Laboratories Ltd.) for 2 h at room temperature. After washing the sections again, they were reacted with 0·3 % avidin-biotin–peroxidase complex (Vector ABC kit; Vector Laboratories Ltd.) for 1 h at room temperature. To observe the antigen–antibody reaction, the sections were reacted with 3,3′-diaminobenzidine (Vector Laboratories Ltd.). After dehydration with ethanol at sequential concentrations, the sections were covered with glass coverslips and observed under a Leica DM5500B microscope (Leica Microsystems, GmbH, Wetzlar, Germany).

### Western blotting

Brain tissue was lysed with radioimmunoprecipitation assay buffer, and proteins were quantified using the BCA Protein Assay Kit (Sigma-Aldrich). Equal amounts of protein were separated by SDS-PAGE. The resolved proteins were transferred onto polyvinylidene difluoride membranes. The membrane was blocked with 5 % skim milk for 1 h and then incubated overnight with the primary antibody diluted in 1 % bovine serum albumin solution at 4°C. The membrane was then incubated with the secondary antibody at room temperature for 2 h. The primary antibodies, PSD-95 (MA1045, Invitrogen, Carlsbad, CA, USA, 1:1000), SYP (ab14692, Abcam, Cambridge, UK, 1:2000), NMDAR 2B (MAB5778, Millipore, 1:1000), BDNF (sc-546, Santa Cruz Biotechnology, 1:1000), and p-ERK (9101S, Cell Signaling Technology, Danvers, MA, USA, 1:1000), ERK (9102S, Cell Signaling Technology, 1:1000), *β*-actin (CSB-PA000350, Cusabio, Houston, TX, USA), and the secondary antibody, anti-rabbit horseradish peroxidase -linked antibody, were obtained from Cell Signaling Technology. The protein bands obtained were measured using an enhanced chemiluminescence detection system (GE Healthcare, Little Chalfont, UK) and Amersham Imager 680 (GE Healthcare), and the bands were analysed using the ImageJ software (National Institutes of Health, Bethesda, MD, USA). The target protein expression levels were calculated using the quantified values of *β*-actin as a reference.

### Statistics

Statistical analysis of data of experiments was performed using Kruskal–Wallis *H* test followed by post hoc Dunn’s test in GraphPad Prism 9.5.1 (GraphPad Software), and the significance between groups was set at *P* < 0·05.

## Results

### Mixture of tomato ethanolic extract and lemon ethanolic extract synergistically improved the cognitive function in aged mice

To investigate the effect of TEE and LEE on the cognitive function of mice, TEE, LEE or their mixture was orally administered and the novel object recognition test was performed after 8 weeks of administration (Fig. 1(a)). The discrimination index, which is used to evaluate animal performance in the novel object recognition test, was significantly lower in the vehicle-treated aged mice than in the vehicle-treated young mice. Although this index tended to be higher in the mice treated with TEE or LEE than in the vehicle-treated aged mice, it was significantly higher in the mice treated with the TEE + LEE mixture than in the vehicle-treated aged mice (Fig. 1(b)). Oral administration of TEE, LEE or their mixture did not significantly affect the body weight of the mice (Fig. 1(c)). These findings suggest that the TEE + LEE mixture synergistically improves the cognitive function of aged mice compared with treatment with TEE or LEE alone.

### Tomato ethanolic extract *+* lemon ethanolic extract mixture synergistically increased the number of doublecortin-positive cells in the hippocampus of aged mice

**Fig. 2.**
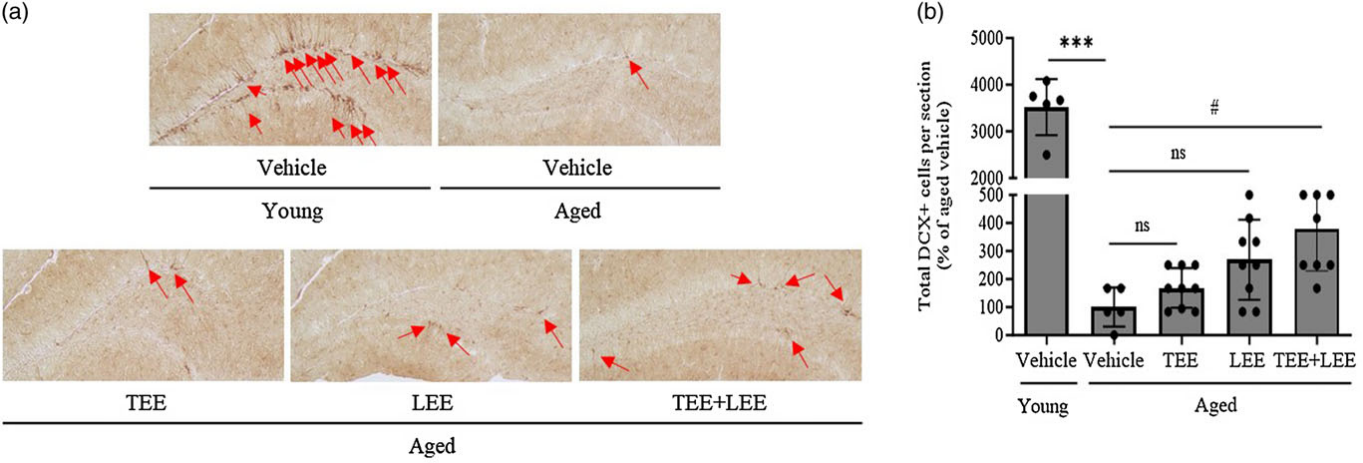
Oral administration of tomato (TEE) and lemon ethanolic extract (LEE) synergistically increased hippocampal neurogenesis in aged mice. Dentate gyrus (DG) neurogenesis was quantified by doublecortin (DCX) immunostaining. (a) Representative images of DCX+ cells in the hippocampal region. Arrows indicate DCX+ cells, and (b) the total number of DCX+ cells in the DG are quantified in the graph. (young + vehicle, *n* 5 mice; aged + vehicle, *n* 5 mice; aged + TEE, *n* 9 mice; aged + LEE, *n* 9 mice; aged + TEE and LEE, *n* 9). Each bar represents the mean ± se for each group. Statistical significance was determined by Kruskal–Wallis *H* test followed by post hoc Dunn’s test. ***P < 0·001 *v*. the young vehicle group. ^#^ *P* < 0·05 *v*. aged vehicle group.

To investigate hippocampal neurogenesis after extract administration in the aged mice, proliferating neuronal progenitors in the DG were assessed by immunostaining of DCX, a marker for neurogenesis. The number of DCX+ cells in the tissue sections from the hippocampus significantly decreased in aged mice compared with that in young mice (Fig. 2(a) and (b)). In TEE or LEE alone-treated group of aged mice, the number of DCX+ cells tended to increase, whereas in the TEE + LEE treatment synergistically increased the number of DCX+ cells compared with the vehicle in aged mice (Fig. 2(a) and (b)). These results suggest that the oral administration of a mixture of TEE and LEE has a synergistic effect on increasing the number of DCX+ cells in the hippocampus.

### Mixture of tomato ethanolic extract and lemon ethanolic extract synergistically increased synaptic protein levels in the mouse hippocampus

**Fig. 3.**
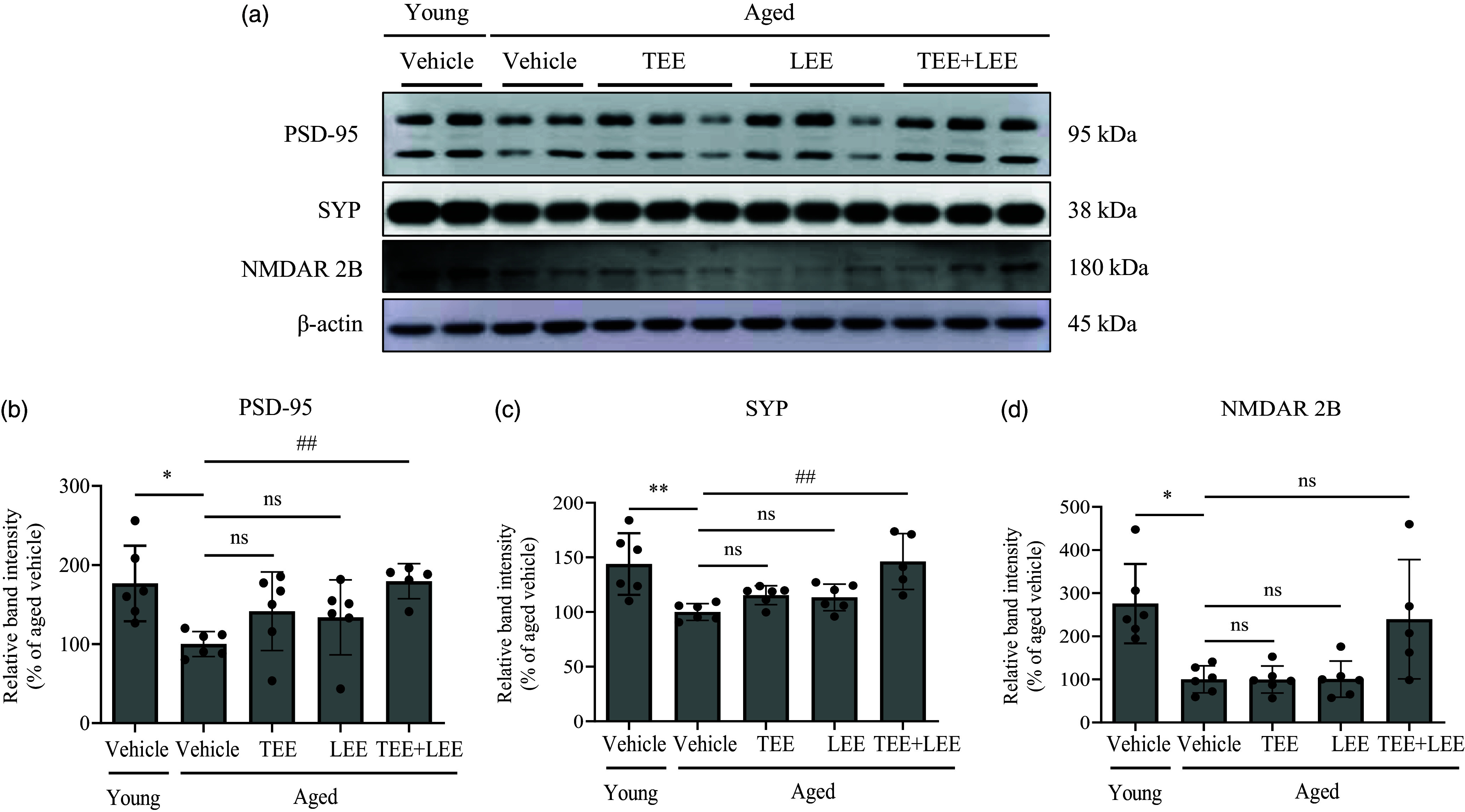
Oral administration of the tomato (TEE) and lemon ethanolic extract (LEE) mixture synergistically increased Synaptic Protein expression in aged mice. The expression of postsynaptic density protein 95 (PSD-95), synaptophysin (SYP) and *N*-methyl-d-aspartate receptor 2B (NMDAR 2B) was assessed by western blotting. (a) Protein bands of young and aged mice treated with TEE, LEE and their mixture are shown. The relative protein expression of (b) PSD-95, (c) SYP and (d) NMDAR 2B was analysed using ImageJ, and the band intensity was normalised to *β*-actin levels. (young + vehicle, *n* 6 mice; aged + vehicle, *n* 6 mice; aged + TEE, *n* 6 mice; aged + LEE, *n* 6 mice; aged + TEE and LEE, *n* 5). Each bar represents the mean ± se for each group. Statistical significance was determined by Kruskal–Wallis *H* test followed by post hoc Dunn’s test. *P < 0·05, **P < 0·01, *v*. young vehicle group. ^##^ *P* < 0·01 *v*. aged vehicle group.

To evaluate the changes in synaptic plasticity, we examined the expression levels of the synaptic plasticity protein markers PSD-95, SYP and NMDAR 2B in the hippocampus by western blotting. The expression of PSD-95 significantly decreased in aged mice compared with that in young mice. In aged mice, TEE or LEE alone treatment did not lead to alterations in PSD-95 expression compared with vehicle treatment. However, the TEE + LEE treatment significantly increased its expression compared with the vehicle treatment in aged mice (Fig. 3(a) and (b)). SYP expression significantly decreased in aged mice compared with that in young mice. In aged mice, the TEE or LEE alone treatment did result in a significant increase in SYP expression compared with the vehicle treatment in aged mice. Interestingly, TEE + LEE treatment not only significantly increased SYP expression compared with the vehicle treatment in aged mice but also showed significant differences compared with TEE or LEE alone treatment (Fig. 3(a) and (c)). The expression of NMDAR 2B significantly decreased in aged mice compared with that in young mice. Compared with the vehicle treatment, the TEE or LEE alone treatment did not lead to alterations in NMDAR 2B expression in aged mice. However, the TEE + LEE treatment resulted in a tendency to increase in NMDAR 2B expression compared with the vehicle treatment in aged mice (Fig. 3(a) and (d)). These results suggest that the oral administration of a mixture of TEE and LEE synergistically increases synaptic plasticity protein levels in the hippocampus.

### Tomato ethanolic extract *+* lemon ethanolic extract mixture synergistically increased mature brain-derived neurotrophic factor level in the mouse hippocampus

**Fig. 4.**
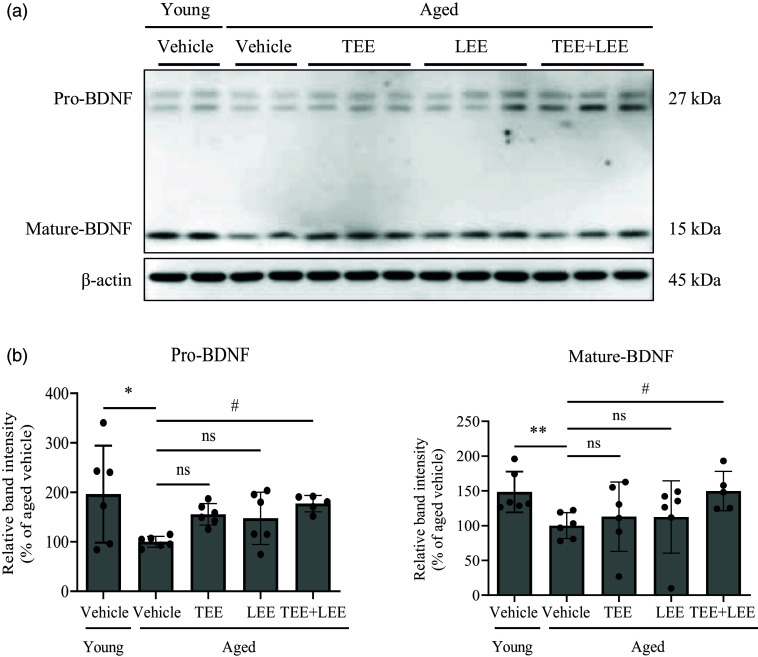
Oral administration of a tomato (TEE) and lemon ethanolic extract (LEE) mixture increased brain-derived neurotrophic factor (BDNF) levels in the hippocampus of aged mice. Hippocampal brain-derived neurotrophic factor (BDNF) levels were measured by western blotting. (a) The bands shown are from young and aged mice treated with TEE, LEE and their mixture. (b) The relative protein expression of pro-BDNF and mature BDNF was analysed using ImageJ, and band intensity was normalised to *β*-actin levels. (young + vehicle, *n* 6 mice; aged + vehicle, *n* 6 mice; aged + TEE, *n* 6 mice; aged + LEE, *n* 6 mice; aged + TEE and LEE, *n* 5). Each bar represents the mean ± se for each group. Statistical significance was determined by Kruskal–Wallis *H* test followed by post hoc Dunn’s test. *P < 0·05, **P < 0·01, *v*. young vehicle group. ^#^ *P* < 0·05 *v*. aged vehicle group.

To elucidate the mechanism underlying cognitive enhancement, BDNF level in the hippocampus of mice was analysed by western blotting. The hippocampus pro-BDNF level significantly decreased in aged mice compared with that in young mice. The TEE treatment significantly increased the pro-BDNF level compared with the vehicle in aged mice. However, The LEE treatment did not significantly increase the pro-BDNF level compared with the vehicle in aged mice. The TEE + LEE treatment synergistically increased the pro-BDNF level compared with the vehicle in aged mice. Similarly, the hippocampal mature BDNF level significantly decreased in aged mice compared with that in young mice. In aged mice, the TEE or LEE alone treatment did not significantly increase mature BDNF level compared with the vehicle in aged mice. However, the TEE + LEE mixture-treated group showed a synergistic increase in mature BDNF level compared with the vehicle group of aged mice (Fig. 4(a) and (b)). These results indicate that the oral administration of the TEE + LEE mixture synergistically increases the mature BDNF level compared with single administration.

### Tomato ethanolic extract *+* lemon ethanolic extract mixture additively activated the tropomyosin receptor kinase B / extracellular signal-regulated kinase signalling pathway in the mouse hippocampus

**Fig. 5.**
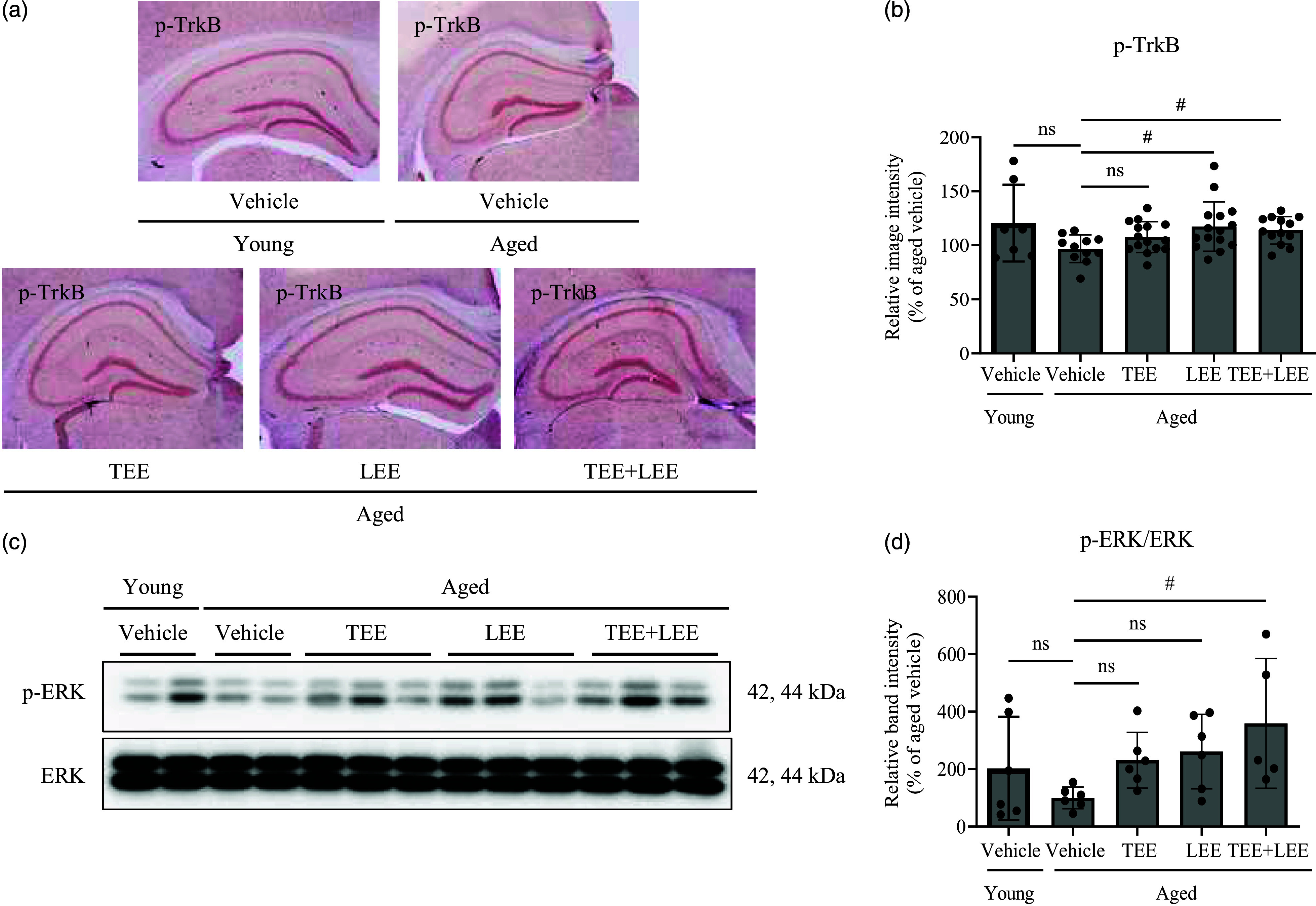
Tomato (TEE) and lemon ethanolic extract (LEE) increased tropomyosin receptor kinase B (TrkB) and extracellular signal-regulated kinase (ERK) phosphorylation in the hippocampus of aged mice. Oral administration of TEE and LEE activated the TrkB/ERK signalling pathway in the hippocampus of aged mice. Phosphorylation of BDNF receptor was quantified by p-TrkB immunostaining. (a) Representative images of TrkB+ cells in the hippocampal region. Arrows indicate TrkB+ cells, and (b) the total number of TrkB+ cells in the DG is quantified in the graph. (young + vehicle, *n* 5 mice; aged + vehicle, *n* 4 mice; aged + TEE, *n* 4 mice; aged + LEE, *n* 5 mice; aged + TEE and LEE, *n* 5). Each bar represents the mean ± se for each group. (c) Changes in the expression of phosphorylated ERK in the mouse hippocampus were analysed by western blotting. (d) The bands shown are the four representative bands from each group. The relative protein expression of phospho-ERK was analysed using ImageJ software. The intensity of the bands was normalised to that of total ERK. (young + vehicle, *n* 6 mice; aged + vehicle, *n* 6 mice; aged + TEE, *n* 6 mice; aged + LEE, *n* 6 mice; aged + TEE and LEE, *n* 5). Each bar represents the mean ± se for each group. Statistical significance was determined by Kruskal–Wallis *H* test followed by post hoc Dunn’s test. ^#^ *P* < 0·05 *v*. aged vehicle group.

To investigate whether the TEE + LEE mixture induces TrkB/ERK phosphorylation in the hippocampus, the phosphorylation of TrkB and ERK was investigated by immunohistochemistry and western blotting. The phosphorylation of TrkB in hippocampal tissue sections tended to be lower in aged mice than in young mice; this difference was not significant (Fig. 5(a) and (b)). In the TEE alone-treated group of aged mice, phosphorylation of TrkB tended to increase, whereas in the LEE or TEE + LEE treatment groups, TrkB phosphorylation was significantly increased compared to in the vehicle in aged mice (Fig. 5(a) and (b)). In the hippocampus of aged mice, ERK phosphorylation tended to decrease compared with that in the hippocampus of young mice; this difference was not significant. However, the TEE- and LEE-treated groups showed a tendency for increased ERK phosphorylation compared with the vehicle group of aged mice. In particular, the TEE + LEE mixture treatment additively increased ERK phosphorylation compared with the vehicle in aged mice (Fig. 5(c) and (d)). These results suggest that the TEE + LEE mixture additively activates the TrkB and ERK pathway compared with a single administration.

## Discussion

In this study, we demonstrated the mechanism underlying the synergistic effect of the TEE + LEE mixture on hippocampal cognitive function, neurogenesis and synaptic plasticity. We suggest that the combination of TEE and LEE leads to enhanced activation of the BDNF/TrkB/ERK signalling pathway, which in turn promotes neurogenesis and synaptic plasticity in the hippocampus and improves cognitive function. This mechanism provides a possible explanation for the observed synergistic effect of the TEE + LEE mixture on cognition enhancement.

### Dual mechanisms of cognitive function improvement by tomato and lemon extracts

There are several mechanisms by which cognitive function can be enhanced, and two of the most well-known ones are through protective effects by antioxidants^(30)^ and neurogenesis^(31)^. Antioxidants, such as vitamin E and flavonoids, found in tomatoes and lemons, can protect brain cells from oxidative stress and damage caused by free radicals^(32)^. Oxidative stress and free radical damage have been linked to several neurological disorders including Alzheimer’s disease, Parkinson’s disease and age-related cognitive decline^(33)^. Previously, our group reported the synergistic effects of TEE and LEE on the enhancement of cognitive function, probably mediated by their cytoprotective effects against oxidative stress^(29)^. In addition to our previous study, herein, we suggested another mechanism underlying the synergistic effect of the TEE + LEE mixture on hippocampal cognitive function, neurogenesis and synaptic plasticity. We demonstrated that the combination of TEE and LEE leads to an enhanced activation of the BDNF/TrkB/ERK signalling pathway, which in turn promotes neurogenesis and synaptic plasticity in the hippocampus and improves cognitive function in aged mice. Therefore, this mechanism provides an additional possible explanation for the observed synergistic effect of the TEE + LEE mixture on cognition enhancement in aged mice.

### Possible effective ingredients in tomato and lemon extracts

In a series of previous studies, it has been suggested that tomato and lemon extracts may have cognitive benefits through antioxidant effects and increase of neurogenesis and synaptic plasticity. For example, a study conducted in a mutant tau (P301L) transgenic Alzheimer’s disease model mice revealed that the supplementation of lycopene, a carotenoid found in tomatoes, improved cognitive performance by reducing oxidative stress^(34)^. Lycopene is also known to increase the number of newly generated cells in the hippocampus of amyloid *β*-induced Alzheimer’s disease rats^(35)^. Similarly, lemon essential oil improves cognitive function in amyloid precursor protein/presenilin-1 double transgenic Alzheimer’s disease model mice with memory impairments through the elevation of BDNF, PSD-95 and SYP^(28)^. Another study has shown that hesperidin, which is a flavonoid found in lemons and other citrus fruits, can restore neurogenesis in the hippocampus of mice with Alzheimer’s disease and improve memory function through the activation of AMP-activated protein kinase/BDNF/TrkB/CREB signalling^(36)^. Although some studies have investigated the individual effects of tomato or lemon extracts on cognitive function, there is a lack of studies specifically examining the synergistic effects of the mixture of tomato and lemon extracts on cognitive function in animals, except our previous study, wherein the protective effects of the mixture of tomato and lemon extracts have been shown^(29)^. Therefore, since there have been no studies about whether a combination of these extracts would have synergistic effects on neurogenesis in animals, we herein investigated whether the mixture of TEE and LEE has a synergistic effect on neurogenesis, and interestingly, demonstrated that the TEE + LEE mixture has a synergistic effect on neurogenesis compared with TEE or LEE alone treatment, by investigating DCX-positive cells and increase of BDNF. This finding suggests that the TEE + LEE mixture may have a greater effect in promoting the generation of new neurons than either extract alone.

### Improvement of synaptic plasticity by tomato and lemon extracts

Some evidence suggests that tomatoes or lemons may have beneficial effects on synaptic plasticity. A previous study found that lycopene supplementation attenuated lipopolysaccharide-induced neuronal damage and synaptic dysfunction in mice by increasing the expression of synaptic protein PSD-95, suggesting a potential improvement in synaptic plasticity^(37)^. Similarly, a previous study demonstrated that TEE increased PSD-95 expression by stimulating the BDNF/ERK/CREB signalling pathway in the hippocampus of aged mice^(25)^. Another study suggested that hesperetin, a flavonoid present in lemons, increased the expression of PSD-95 by stimulating the CREB signalling pathway in the hippocampus of lipopolysaccharideinduced neuroinflammation model in mice^(38)^. In particular, there has been no evidence of the effects of tomatoes or lemons on NMDAR expression, which is another marker of synaptic plasticity^(13)^. NMDAR is an important glutamate receptor for synapse formation and learning and memory function. Phosphorylation of NMDAR by protein kinases such as protein kinase C and Calcium/calmodulin-dependent protein kinase II has been shown to modulate NMDAR activity and trafficking^(39)^. Additionally, the expression of NMDAR can be regulated by transcription factors such as CREB, which is activated by BDNF and other signalling molecules^(40)^. However, in ageing, changes in NMDA receptor subunit expression and function have been reported in various studies. In aged mice, there was no decrease in NMDAR 1, but there was a decrease in NMDAR 2B^(41,42)^. In this study, we demonstrated that NMDAR 2B, PSD-95 and SYP are significantly decreased in aged mice and the TEE + LEE mixture synergistically increased the expression of NMDAR 2B, PSD-95 and SYP, whereas neither TEE nor LEE significantly increased their expression alone in the doses of our experiment. However, the TEE + LEE mixture did not a significant increase in NMDAR 1 and 2A expression compared with the vehicle treatment in aged mice (data not shown). Although we still do not know the exact underlying mechanism for the synergistic increase in NMDAR 2B, PSD-95 and SYP expression levels, our results suggest that their synergistic increases may be involved in the synergistic cognitive-enhancing effects of a mixture of tomatoes and lemons.

### Enhancement of brain-derived neurotrophic factor pathway by tomato and lemon extracts

The BDNF/ERK signalling pathway plays a crucial role in regulating cognitive function and neurogenesis^(43)^. Recently, oral administration of tomato extract was found to improve cognitive function in aged mice by increasing hippocampal neurogenesis and synaptic plasticity via the BDNF/ERK/CREB signalling pathway^(25)^. Supplementation of lycopene also has been reported to improve cognitive function by regulating BDNF and Nrf2/NF-kB signals in the hippocampus^(44)^. The inhalation of lemon essential oil regulates synaptic plasticity and cognitive function by stimulating the BDNF/ERK/Akt signalling pathway^(28)^. Additionally, it has been suggested that vitamin C, which is abundant in lemons, may have a protective effect on the brain and potentially improve cognitive functions^(45)^. Our results showed that pro-BDNF and mature BDNF levels decreased, but the phosphorylation of ERK was not significantly different in the aged mouse model. Interestingly, however, the combination of tomato and lemon extract synergistically increased mature BDNF levels compared with single extract treatment, and mature BDNF and phosphorylation of ERK significantly increased. The finding that tomato and lemon extract mixture increased the BDNF/ERK signalling pathway suggests that this combination may have the potential to enhance neuroplasticity and cognitive function in ageing populations. The active ingredients of tomatoes and lemons, including lycopene^(46)^, vitamin C^(47)^, citric acid^(48)^ and flavonoids^(49)^, are also known to be effective in improving cognitive function and memory through the regulation of BDNF and alleviating other neurological diseases, such as Alzheimer’s disease, schizophrenia, Parkinson’s disease and depression^(50)^. As BDNF alleviates several nerve-related diseases, it is possible that tomato and lemon extracts could also be applied to various nerve diseases. However, further research is needed to determine the potential therapeutic effects of tomato and lemon extracts against neurological disorders.

### Limitations of the study

The results of our study are subject to limitations, particularly in terms of our understanding of the specific constituents of tomatoes and lemons that may be responsible for potential synergistic effects. Furthermore, our study lacks results concerning whether the components of tomato and lemon extracts influence cognitive function by crossing the blood-brain barrier or modulating the gut-brain axis. In future studies, we will analyse the active constituents of tomatoes and lemons and determine whether these components pass through the gut-brain axis or directly via the blood-brain barrier.

### Conclusion

In the present study, we showed that TEE and LEE exert synergistic effects to enhance cognitive function, neurogenesis and synaptic plasticity in aged mice and elucidated the signalling pathways. Our results showed that the administration of the TEE + LEE mixture increased the phosphorylation of TrkB and ERK in the hippocampus possibly via BDNF. Therefore, in conclusion, a mixture of tomato and lemon extracts has a synergistic effect in improving cognitive function in aged mice, which provides implications for developing new treatments or dietary interventions to improve cognitive function in ageing populations.
